# Viruses and Endogenous Retroviruses as Roots for Neuroinflammation and Neurodegenerative Diseases

**DOI:** 10.3389/fnins.2021.648629

**Published:** 2021-03-12

**Authors:** Christine Römer

**Affiliations:** Max Delbrück Center for Molecular Medicine in the Helmholtz Association, The Berlin Institute for Medical Systems Biology, Berlin, Germany

**Keywords:** HERV, LINE, virus, neurodegeneration, neuroinflammation

## Abstract

Many neurodegenerative diseases are associated with chronic inflammation in the brain and periphery giving rise to a continuous imbalance of immune processes. Next to inflammation markers, activation of transposable elements, including long intrespersed nuclear elements (LINE) elements and endogenous retroviruses (ERVs), has been identified during neurodegenerative disease progression and even correlated with the clinical severity of the disease. ERVs are remnants of viral infections in the human genome acquired during evolution. Upon activation, they produce transcripts and the phylogenetically youngest ones are still able to produce viral-like particles. In addition, ERVs can bind transcription factors and modulate immune response. Being between own and foreign, ERVs are reviewed in the context of viral infections of the central nervous system, in aging and neurodegenerative diseases. Moreover, this review tests the hypothesis that viral infection may be a trigger at the onset of neuroinflammation and that ERVs sustain the inflammatory imbalance by summarizing existing data of neurodegenerative diseases associated with viruses and/or ERVs.

## Introduction

Viruses have long been linked with diseases of the nervous system. Several viruses, including human α-herpesvirus types 1, 2, and 3 (HHV-1 and HHV-2, known as herpes simplex viruses, and HHV-3, known as varizella zoster virus), human cytomegalovirus (CMV), human immunodeficiency virus (HIV), Epstein–Barr virus (EBV), Ebola virus, and rabies virus are capable of reaching the central nervous system (CNS) (Dando et al., 2014). Often, particular viral nucleic acids or proteins are found in the brain, cerebrospinal fluid (CSF), or peripheral blood of patients with a certain neurological disease. For example, HHV-3 and HHV-6 are present in the CSF (Mancuso et al., 2007; Alvarez-Lafuente et al., 2008), coronaviruses in the CNS of multiple sclerosis (MS) patients (Burks et al., 1980), and HIV and human T-cell leukemia virus-1 (HTLV-1) in the brains of amyotrophic lateral sclerosis (ALS) patients (Verma and Berger, 2006). HHV-6A DNA and transcripts, in turn, are increased in the brains of Alzheimer’s disease (AD) patients and closely correlate with neuronal loss (Readhead et al., 2018). Tracing neuropathologies to viral infections can, however, be challenging. This holds particularly true when the virus becomes “slower” or “latent” following acute infection (Sigurdsson, 1954; Murphy and Yunis, 1976; Steiner et al., 2007; Kennedy and Cohrs, 2010; Shu et al., 2015; Rodriguez et al., 2020). The tremendous research from the beginning of the HIV pandemic has greatly enhanced evidence and understanding of this slow action of viruses in the CNS (Garcia et al., 1999). Important to consider also is the long-term risk from accumulated infections during a lifetime that might lead to a cumulative and individual risk of developing neuropathology, such as stroke and dementia (Almeida and Lautenschlager, 2005; Ruprecht et al., 2006; Tai et al., 2009; Sico et al., 2015).

More recent research has shown that viruses, such as HIV, EBV, CMV, influenza, herpesviruses, and HTLV-1 can activate viral sequences originating from retroviral infections in the distant past of human evolution that have been incorporated into the human genome (Nellaker et al., 2006; Toufaily et al., 2011; Young et al., 2012; Leboyer et al., 2013; Li et al., 2014; Kury et al., 2018). While their ability to express viral products is mostly lost, some of these endogenous retroviruses (ERVs) have evolved to play important roles in physiological processes, such as placentation, early human embryogenesis, neurodevelopment, and immune response regulation (Kammerer et al., 2011; Wang et al., 2014, 2020; Chuong et al., 2016; Romer et al., 2017; Xue et al., 2020). Activation of ERVs, such as by exogenous viruses or environmental factors, can contribute to a multitude of neurodevelopmental, neurodegenerative, and neuroinflammatory disorders (Balestrieri et al., 2019; Gruchot et al., 2019, 2020; Tam et al., 2019; Evans and Erwin, 2020; Groger et al., 2021), including HIV-associated neurodegenerative disorder (HAND), AD, MS, ALS, schizophrenia, stroke, and neuropathogenesis of severe acute respiratory syndrome coronavirus-2 (SARS-CoV-2) as well as to accelerated neurological decline in aging.

This review highlights the interplay between endogenous viruses and retroelements, on the one hand, and exogenous viruses, on the other hand, and aims at revealing underlying mechanisms in aging, and neurodegenerative and neuroinflammatory diseases summarizing recent advances in this field.

## Viral Infection of the Central Nervous System

**FIGURE 1:**
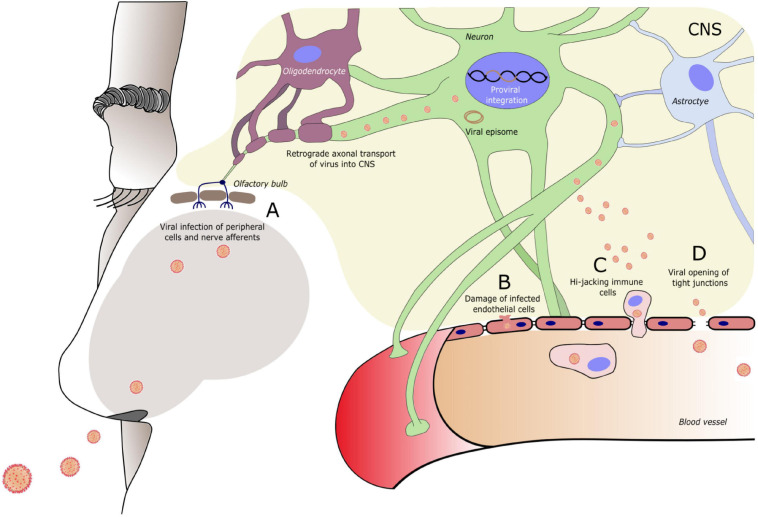
Entry routes of exogenous viruses into the central nervous system (CNS). Virus can enter the CNS via **(A)** Peripheral nerve terminals (in this example of the olfactory nerve) and retrograde axonal transport. **(B)** Infection of and damage to endothelial cells of the blood brain barrier (BBB). **(C)** Infection of circulating immune cells that travel across the BBB. **(D)** Modulation of tight junction molecules of the BBB, increasing BBB permeability. A specific entry route can be typical for a specific virus, however, often more than one route is used. Entry route may vary during the course of infection (e.g., BBB damage in conditions of high viremia) and new CNS entry mechanisms can follow when the viral genome sustains mutational changes. A neurotrophic virus often infects specific cell types within the CNS. Reaching the CNS enables the virus to circumvent the peripheral immune response. To evade also the CNS immune response, viruses may enter a dormant state forming a viral episome or integrating into the host cell DNA.

The central nervous system (CNS) is not a common target organ for viruses. It is neither easily accessible nor as advantageous in terms of contagiousness and successful viral transmission to new hosts as the respiratory or gastrointestinal tract. Shielded by the meninges, CSF, and blood brain barrier (BBB), the CNS is immunologically unique and privileged (Louveau et al., 2015). Although the CNS itself is armed with an array of immunological mechanisms, including support from the periphery, it may be considered as a sanctuary where viral replication occurs despite a complete viral suppression in the peripheral blood. This has been shown, for example, for the HIV (Walker et al., 2008). In addition to viruses with neurotropism, only minor mutations may be sufficient to create viruses that can access the CNS via various routes (Wiley, 2020). Permeability of the BBB may be increased by high viremia accompanied by elevated cytokine levels and also by direct interaction with tight junction proteins (Toborek et al., 2005; Chai et al., 2014). Viruses can infect endothelial cells of the BBB, allowing viral replicates to be released into the CNS (Verma et al., 2009; Fletcher et al., 2012), while infection of leukocytes or monocytes by, for example, HIV and SARS-CoV-2, that pass BBB physiologically, provides a “trojan-horse” mechanism to enter the CNS (Larochelle et al., 2011; Takeshita and Ransohoff, 2012; Bostanciklioglu, 2020). Attention is currently drawn to the CNS invasion through retrograde neuronal transport of infected peripheral nerve afferents, as SARS-CoV-2 and other coronaviruses are associated with CNS entry via the olfactory pathway, a mechanism that has been also described for other viral families such as influenza A virus, rabies virus, and herpesviruses (van Riel et al., 2015), and other peripheral nerves, for example, the sciatic nerve and vagus nerve (Ren and Racaniello, 1992; Ohka et al., 1998; Guadarrama-Ortiz et al., 2020; Liu et al., 2020). Figure 1 depicts the mechanisms of viral entry into the CNS.

Once in the CNS, acute infections present with encephalitis, myelitis, or viral meningitis. Generally, virus-triggered immune reaction is limited in time and ends with the virus being combated; however, certain neurotropic viruses can continue to elicit progressive damage on brain structure, function, and cognition long after the clearance of virus from the peripheral blood. In addition to this type of chronic infections, viruses can enter a latent (dormant) phase, interrupted by occasional full awakening of the virus. Sometimes, the same virus can contribute to both. This is the case for the HIV (Rodriguez et al., 2020), measles morbillivirus (Murphy and Yunis, 1976), HHV-1 (Shu et al., 2015), and HHV-3, to name a few (Steiner et al., 2007; Kennedy and Cohrs, 2010). The high worldwide seroprevalence of some of these viruses, such as that of HHV-1 and HHV-2 being around 90% (Wald and Corey, 2007), indicates that facilitating factors must exist that ultimately decide upon disease development. In consideration are comorbidities such as traumas to latently infected neurons (Zhang et al., 2013), immune-depriving conditions such as AIDS (Rodriguez et al., 2020), leukemia (Koskenvuo et al., 2008; Lancman et al., 2020), or stroke-induced immunodepression (Deroux et al., 2012; Hetze et al., 2013; Romer et al., 2015; Bertrand et al., 2019), and cumulative infectious burden (Sico et al., 2015), and also environmental factors (Liu et al., 2013; Brutting et al., 2018; Mueller et al., 2018; Del Re and Giorgi, 2020). The role of aging as a facilitating factor and the interplay with ERVs are discussed in detail below.

## Endogenous Retroviruses and Retroelements

Exogenous retroviruses from which ERVs originate, like other retroviruses, contain single-stranded (ss) anti-sense RNA and RNA reverse polymerase to generate double-stranded DNA (dsDNA). With the help of the retroviral integrase, this DNA copy may become endogenized into the host genome, essentially when infecting gametes (germ cells) with chromosomal insertion sites that will allow the birth of viable offspring over generations. Over the evolution, this type of infections and endogenizations of retroviruses have occurred multiple times (Kozak, 1985). Gradually, ERVs become non-infectious, lose the ability to exit the host cell, and adopt the nature of transposable elements. ERV sequences become transpositionally inactive, mutated, degraded, and epigenetically silenced as part of the host control in protection of genome stability. ERV sequences take up about 8% of the modern human genome (Gannet, 2019). ERV families that have been less prone to be degraded by the host, such as human ERV H (HERV-H) and HERV-K, have shaped the evolution and complexity of innate and adaptive immune pathways (Villarreal, 2011; Chuong et al., 2016, 2017). Regulation mechanisms to control the HERV activity, mainly via epigenetics (for example, cytosine methylation) form the basis for proper host–HERV interaction in controlling vital processes (Lavie et al., 2005; Turelli et al., 2014). The Krüppel-associated box domain (KRAB)-associated protein-1 (KAP1)-mediated silencing continues to be the key mechanism of ERV control in adult brain (Fasching et al., 2015). KAP1 deletion during brain development is lethal, and heterozygous deletion of KAP1 causes behavioral changes resembling those observed in human psychiatric conditions associated with HERV upregulation (Jakobsson et al., 2008).

Long interspersed nuclear elements (LINEs) are a group of non-LTR (long terminal repeat) retroelements that compose up to 21% of the human genome (Gannet, 2019). LINE-1 elements are a major source of structural polymorphisms in humans (Hancks and Kazazian, 2012). Higher LINE-1 activity is characteristic to brain areas of adult neurogenesis, in particular to the hippocampal dentate gyrus (Baillie et al., 2011; Kurnosov et al., 2015; Bachiller et al., 2017) and to human neural progenitor cells (Coufal et al., 2009). LINE-1 insertions often locate at neuronal genes, and LINE-1 activity can initiate neuronal differentiation (Muotri et al., 2005). Hippocampal LINE-1 activity and genomic mosaicism are involved in cognitive processes such as memory formation (Bachiller et al., 2017).

Alu family is the most common member of the short interspersed nuclear elements (SINEs) and accounts for about 13% of the human genome (Gannet, 2019). Alu elements are actively transposing. However, they do not encode a functional reverse transcriptase protein and therefore rely on the machinery of other retroelements, especially LINEs (Wei et al., 2001; Dewannieux et al., 2003). Alu elements are involved in neurogenesis, brain connectome development, and in shaping cognition networks (Mehler and Mattick, 2007; Baillie et al., 2011; Bedrosian et al., 2016).

HERVs, LINEs, and Alus regulate gene expression networks at multiple levels, providing a rich pool for RNA diversification (Lev-Maor et al., 2003; Laperriere et al., 2007; Fujii, 2010; Gim et al., 2014; Goke and Ng, 2016), functioning as promoters and enhancers (Norris et al., 1995; Shen et al., 2011; Lee, 2012; Wu et al., 2013; Romer et al., 2017), and coordinating 3D genomic arrangements via topologically associated domains (Dixon et al., 2012; Zhang et al., 2019). These elements contribute significantly to defining neurobiological processes, including neuronal mosaicism and shaping brain development (Coufal et al., 2009; Baillie et al., 2011; Richardson et al., 2014; Bodea et al., 2018). Alteration of these networks are associated with neurodevelopmental, neurodegenerative, neuroinflammatory, and autoimmune diseases. HERVs, LINE, and Alu elements are subject to a multitude of environmental factors and xenobiotics, which can activate normally well-controlled HERV expression, such as hypoxia (Brutting et al., 2018), drugs (aspirin, caffeine, and valproic acid) (Diem et al., 2012; Liu et al., 2013), and hormones (Norris et al., 1995; Mueller et al., 2018). Also, LINE-1 retroelement activity is sensitive to a multitude of factors including social isolation stress, heavy metals, and anti-inflammatory and psychoactive drugs (Del Re and Giorgi, 2020). Together, (H)ERVs, LINE, and Alu elements regulate early human embryogenesis, neurodevelopment, neural diversity, and plasticity. All three are subject to a number of environmental factors, affecting a healthy brain.

## Interplay Between Endogenous and Exogenous Viruses

Inherent ERVs and exogenous viruses, being distant relatives, share common mechanisms but can also be opponents. When viruses first try to enter a cell, HERV, coming from inside the host genome, can provide protection by blocking the cellular receptors relevant for the exogenous retrovirus entry (Spencer et al., 2003). ERVs can protect against exogenous retroviral infections by receptor interference if both viruses share the specificity of the env glycoprotein (Weiss et al., 1985). Substantial similarity between HERV and exogenous retrovirus, such as that between HERV-K (HML-2) gag and HIV gag, could lead to fusion of viral proteins and production of defective viral particles (Monde et al., 2012). In addition, HERV antisense transcripts can interact with complementary exogenous retrovirus transcripts to block viral replication and generate dsRNA to be recognized as a pathogen-associated molecular pattern (PAMP) by the host immune system (Tang et al., 2012; Shekarian et al., 2017). Sensing PAMPs, such as viral proteins and nucleic acids, and danger-associated molecular patterns (DAMPs) derived from damaged cells, are part of the innate immune response. Cytoplasmic sensors for viral DNA include cyclic GMP-AMP synthase (cGAS), Z-DNA-binding protein 1 (ZBP1), and TLR9 (Rigby et al., 2014; Hayashi et al., 2015; Xia et al., 2016; Sandstrom et al., 2017; Jiao et al., 2020). Viral RNA are sensed by TLR8 (Heil et al., 2004), TLR3, melanoma-differentiated-associated gene 5 (MDA5), ZBP-1 (Gurtler and Bowie, 2013; Jiao et al., 2020), and retinoic acid inducible gene I (RIG-I) (Gurtler and Bowie, 2013). Innate immune response to viral infections leads to pro-inflammatory cytokine, chemokine, and type I interferon (IFN) release to stimulate adaptive immune response, the T lymphocyte-mediated cellular and B lymphocyte-mediated humoral immunity.

Activated innate and adaptive immune system cells both can stimulate ERV transcription (Bannert and Kurth, 2004). Generally, immune reactions are limited in time and cleared by the immune system. However, HERVs are continuously present and, under certain conditions, also continuously active. Aiming at clearing up the infection triggered by HERVs, TLR stimulation can, via IFN release, actually activate HERVs further (Bannert and Kurth, 2004). Dispersed at relevant immune genes, activated HERVs and in particular the polymorphic HERV-K (HML-2) loci, form another layer of immune response regulation (Nexo et al., 2011). Certain HERV insertions function as IFN-inducible enhancers, and type I IFN is one of the main innate immune response products to viral infection (Chuong et al., 2016). Neuroinflammation will awaken and activate HERVs in the human brain (Johnston et al., 2001; Manghera et al., 2015, 2016). In this feedback loop, HERV activity is upregulated by anti-viral immune response through inflammatory mediators and also by epigenetic dysregulation (Manghera and Douville, 2013; Hurst and Magiorkinis, 2015, 2017), leading to chronic stimulation of the immune system (Hurst and Magiorkinis, 2015, 2017; Grandi and Tramontano, 2017; Mameli et al., 2017; Ramasamy et al., 2017). Continuous ERV activation is associated with sustained neuroinflammation and predisposes to neurodegenerative and autoimmune diseases (Nexo et al., 2011).

Activation of ERV transcription can directly be achieved by several exogenous viruses, such as HIV, EBV, CMV, influenza, and herpesviruses, some of which can even induce a self-sustained HERV activation (Nellaker et al., 2006; Young et al., 2012; Leboyer et al., 2013; Li et al., 2014; Kury et al., 2018). Among exogenous retroviruses, HTLV-1 Tax can increase HERV-H, HERV-K, HERV-W, and HERV-E expression in T lymphocytes (Toufaily et al., 2011). HIV transactivator of transcription (Tat) protein can stimulate expression of HERV-K and HERV-W in astrocytes and peripheral blood cells and that of HERV-W also indirectly via TLR4 and proinflammatory cytokine (TNF-α, NF-κB) production (Uleri et al., 2014). Using mimicry, HIV rev, which mediates nuclear export of HIV messenger RNA (mRNA), also mediates the nuclear export of HERV-K mRNA, thereby promoting HERV-K translation (O’Carroll et al., 2020). Exogenous viruses can further facilitate expression of endogenous superantigens, linked in particular with the CNS-affecting autoimmune diseases (Acha-Orbea, 1992). This occurs, for example, between rabies virus and HERV-W (Lafon et al., 1992; Perron et al., 2001; Lafon et al., 2002) and between EBV and HERV-K18 (Sutkowski et al., 2001). In turn, ERVs may assist their exogenous counterparts to escape immune surveillance, repair defects in exogenous retroviruses (Schwartzberg et al., 1985), and facilitate chronic viral replication (Rasmussen, 1997). Also, transcriptionally active ERVs provide a rich pool for recombinational events with exogenous retroviruses. When a host cell is infected by two different viruses, heterologous *trans*-activation can take place where transcription of one virus is initiated by factors produced by the other virus. When ERVs provide envelope glycoproteins to exogenous retroviruses, these could establish a new host cell repertoire and circumvent immune system response (Lusso et al., 1990). A certain degree of epitope similarity between ERV and exogenous retrovirus can lead to a weaker immune response against this virus (Miyazawa and Fujisawa, 1994).

Similar interplay between endogenous retroviruses and exogenous viruses exists in the periphery and may pave way to chronic inflammation. In fact, viruses and endogenous retroviruses have been linked with autoimmune disease pathology, such as systemic lupus erythematosus (Ogasawara et al., 2000; Moon et al., 2004), rheumatoid arthritis (Herve et al., 2002), and diabetes (Levet et al., 2017, 2019). Continued upregulation of HERV-H and HERV-K after the clearance of hepatitis C virus from the peripheral blood of chronic hepatitis C patients was recently associated with higher risks for cancer and autoimmunity in these patients (Tovo et al., 2020).

Peripheral inflammation can reach the brain via transversal of circumventricular organs, peripheral nerves, or through pro-inflammatory cytokine influx upon direct cytokine–endothelial interactions, resulting in reduced BBB integrity (Toborek et al., 2005; Chai et al., 2014). Moreover, peripheral inflammation processes can trigger major neurological events such as stroke via platelet aggregation, hypercoagulation, impaired endothelial function, and thrombosis (Elkind et al., 2020; Oxley et al., 2020).

**FIGURE 2:**
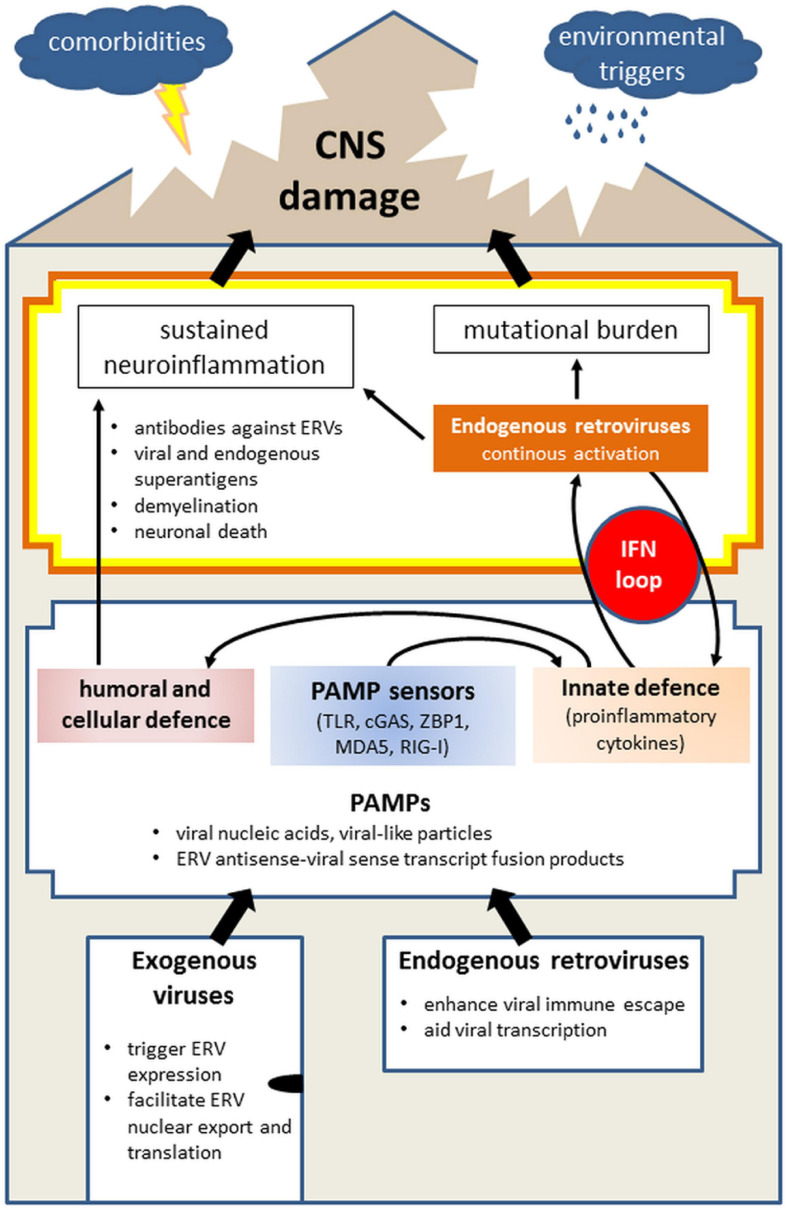
Model of the role of exogenous virus and endogenous retrovirus (ERV) in initiating neuropathologies, illustrating the entry of viruses into the body (“house”) and the presence of ERVs, the physiological immune response on the first level and continuous imbalance on the second, thus leading to damage of the CNS (“roof”). Infections with exogenous viruses often activate the transcription of endogenous retroviruses which are already present in the mammalian genome. This results from disruption of the host-control over ERVs, bordering between own and foreign. Separately and together, activated exogenous viruses and ERVs produce PAMPs, such as viral nucleic acids, viral-like particles, fused transcripts between exogenous virus, and ERV, which are sensed by PAMP sensors (TLR, cGAS, ZBP-1, MDA5, and RIG-I). Collectively, PAMP activation alarms the immune system by initiating innate immune response involving mainly IFN signaling which will trigger the adaptive (humoral and cellular) immunity. Viral infections are generally limited in time as they are combated by the immune system. However, the activation of ERVs and neurotrophic viruses which remain silent yet present within the body, can lead to sustained neuroinflammation via antibodies against ERVs, superantigen formation, demyelination, and neuronal death. In parallel, IFN-mediated innate immune response can further activate ERVs which contain IFN response elements (such as HERV-K), thereby creating an IFN loop. Next to the danger of chronic neuroinflammation, this additionally carries mutational burden for the host, collectively leading to the CNS damage. The probability and scope of the CNS damage is further determined by facilitating factors, including comorbidities and environmental triggers as well as age. Abbreviations: cGAS, cyclic GMP-AMP synthase; CNS, central nervous system; ERV, endogenous retrovirus; IFN, interferon; MDA5, melanoma differentiated associated gene 5; PAMP, pathogen-associated molecular pattern; RIG-I, retinoic acid inducible gene I; TLR, toll-like receptor; ZBP-1, Z-DNA binding protein 1.

Figure 2 draws common mechanisms in the interplay of exogenous and endogenous retroviruses leading to sustained neuroinflammation and subsequent CNS damage.

## Viral and Endogenous Retroviral Associated Pathologies

Table 1 summarizes exogenous viral and endogenous retroviral disorders discussed in the following sections.

**TABLE 1:** Overview of disorders affecting the central nervous system (CNS) associated with an onset mediated by exogenous viruses and/or endogenous retroviruses.

**Disorder**	**Clinical CNS symptoms**	**Associated peripheral pathology**	**CNS targets**	**Associated exogenous viruses**	**Associated endogenous retroviruses**	**Mechanisms**
HIV-associated neurodegenerative disorder (HAND)	Cognitive, motor, and behavioral deficits similar to AD	AIDS	Microglia, astrocytes, and perivascular macrophages, neurons (motor and cortical)	HIV-1 (aggravation by CMV, EBV, HHV-3, and HHV-6)	HERV-K (HERV-E, HERV-T, two ERV9 subgroups)	• Neurotoxic viral products (Tat, Vpr, HIV-1 gp120)
						• Cytokine-induced HERV expression (IL-6, IL-1β, TNF-α, IFN-γ)
						• Viral induction of antibody against HERV-K capsid protein
						• Sustained inflammation
						• Increased pTau, neopterin, neurofilament light and diffuse Aβ plaques
Alzheimer’s disease (AD)	Cognitive deficits (memory loss, learning difficulty, impaired logical thinking, confusion, speech problems, shortened attention span)	Systemic immune activation, and chronic peripheral inflammation	Microglia, hippocampal pyramidal neurons lymphocytes, neuronal, and endothelial cells parahippocampal, inferior frontal and superior temporal gyrus	HHV-1, HHV-6A, HHV-6B, HHV-7, EBV, and CMV	HERV-K, HERV-H, HERV-W, HERV-L, solitary long-terminal repeats (LTRs)	• Extension of inflammation to CNS (immune cells entry to brain via transversal of circumventricular organs, vagus nerve stimulation or pro-inflammatory cytokine influx)
						• Viral RNA sensor MAVS • Gliosis
						• Gliosis
						• Viral activation of HERVs
						• Higher rate of DNA damage and higher expression of pluripotency-related genes
						• HERV fusion products (ARC viral-like capsid protein overexpression)
						• HERV-induced TLR8 activation
						• Progressive neuronal death (PARP1-driven, caspase-independent apoptosis)
						• Dense Aβ plaques, Tau neurofibrillary tangles
Multiple sclerosis (MS)	Progressive physical and cognitive disabilities neurobehavioural deficits, such as weakness, gait unsteadiness, and altered executive functions	Hints for chronic inflammation, association with peripheral neuropathy	B cells, microglia, astrocytes, and macrophages that orchestrate damage to oligodendrocytes	HHV-1, HHV-2, HHV-3, HHV-6, EBV, CMV, JCV	HERV-W (HERV-K, HERV-H)	• Viral activation of HERV-W transcription
						• HERV-W env protein and syncytin expression
						• HERV-W env is a powerful superantigen
						• Syncytin induces neuroinflammation via oxidative stress
						• Stimulate anti-viral response associated with MS pathology by binding TLR4 and CD14
						• Pro-inflammatory (anti-viral) response involving TLR4, CD14, IL-1beta
						• Increase in cellular protein oxidation, inhibition of oligodendrocyte maturation, myelin damage and antagonization of remyelination
Amyotrophic lateral sclerosis (ALS)	Fasciculation, cramps, muscle atrophy, and marked limb weakness	HERV-W env and gag are present in muscle biopsies from ALS patients, linked with macrophage activation and neurogenic atrophy of muscular tissue	HERV activity in prefrontal, sensory, motor, occipital cortex	Weak connection with HIV-1, and HTLV-1	HERV-K	• HERV-K transcription in ALS is stimulated by the TDP-43
						• HERV-K transcription can be also initiated by HIV-Tat protein HERV-K env in cortical and spinal neurons
						• neuronal HERV-K activation is associated with the nuclear translocation of interferon regulatory factor 1 (IRF1)
						• Sustained neuroinflammation with progressive loss of cortical and spinal motor neurons
Schizophrenia spectrum disorders	Psychosis, hallucinations, delusions, apathy and disorganized thinking	Subclinical inflammation	Neurons of prefrontal cortex and (developing) hippocampus	HHV-2, perinatal influenza infection	HERV-W, LINE-1 (HERV-K, HERV-H)	• Impairment of synaptic genes
						• Upregulation of immune response genes
						• Lasting inflammatory dysregulation of the nervous system
Neuropathogenesis of SARS-CoV-2	Dizziness, headache, encephalitis, seizures, intracerebral hemorrhage, and stroke, neuromuscular and autoimmune syndromes	Acute respiratory disease	Viral infection of neurons and glial cells	SARS-CoV-2	none	• Interacts with stress response, vesicle trafficking, lipid metabolism pathways, production of reactive oxygen species, RNA processing, RNA regulation, ubiquitin ligases and mitochondrial activity
						• Impaired lysosomal function combined with inhibition of ubiquitin-proteasome system
						• Protein misfolding and formation of protein aggregates
						• Expected neuroinflammation and neurodegeneration

*A*β, *amyloid* β; *AD, Alzheimer’s disease; AIDS, acquired immunodeficiency syndrome; ALS, amyotrophic lateral sclerosis; CD14, cluster of differentiation 14; CMV, cytomegalovirus; EBV, Epstein-Barr virus; gp120, glycoprotein 120; HAND, HIV-associated neurodegenerative disorder; HERV, human endogenous retrovirus; HHV-1, human alphaherpesvirus-1/herpes simplex virus-1; HHV-2, human alphaherpesvirus-2/herpes simplex virus-2; HHV-3, human alphaherpesvirus-3/varicella zoster virus; HHV-6, human alphaherpesvirus-6; HHV-7,human alphaherpesvirus-7; HIV-1, human immunodeficiency virus-1; HTLV-1, human T-cell leukemia virus-1; IFN-γ, interferon γ; IL, interleukin; IRF1, interferon regulatory factor 1; JCV, John Cunningham virus; LINE-1, long interspersed repetitive element 1; LTR, long-terminal repeat; MAVS, mitochondrial antiviral-signaling protein; MS, multiple sclerosis; SARS-CoV-2, severe acute respiratory syndrome coronavirus-2; Tat transactivator of transcription; TDP-43, trans-activation responsive TAR; DNA-binding protein 43; TLR, toll-like receptor; TNF*-α, *tumor necrosis factor* α; *Vpr, viral protein R*.

### HIV-Associated Neurodegenerative Disorder

HIV infection causes acquired immunodeficiency syndrome (AIDS) affecting multiple systems in the body. One of the complications of HIV infection is the HIV-associated neurodegenerative disorder (HAND) (Navia et al., 1986), which can develop into HIV-associated dementia (Nookala et al., 2017), the most common cause of dementia in young adults (Janssen et al., 1992) with higher prevalence among women (Duarte et al., 2020). HIV is transported to the brain with infected T-lymphocytes and monocytes (Wiley et al., 1986; Takahashi et al., 1996). These long-lived cells are referred to as sources of HIV chronic infection (Nookala et al., 2017). In the brain, HIV infects primarily the immunocompetent cells, perivascular macrophages, and microglia where it replicates (Watkins et al., 1990; Albright et al., 2000). HIV persists in the CNS, causing motor, cognitive, and behavioral deficits, which can be further aggravated by opportunistic infections by CMV, EBV, HHV-3, and HHV-6 (Almeida and Lautenschlager, 2005).

Neurodegeneration characteristic to HAND emanates from chronic inflammation, sustained by activated monocytes, macrophages and astrocytes, and neurotoxic HIV viral proteins (Ghafouri et al., 2006; Kraft-Terry et al., 2010). These include HIV Tat, HIV viral protein R (Vpr), and HIV env glycoprotein gp160 cleaved product gp120. HIV viral proteins induce neuropathology by aberrant calcium signaling, mitochondrial damage, oxidative stress, excitotoxicity, and inflammation (Nookala et al., 2017), collectively leading to neuronal death (Masliah et al., 1992).

Further augmentation of neurodegeneration and neuroinflammation in HAND comes from HIV and infection-induced cytokines’ (IL-6, IL-1β, TNF-α, and IFN-γ) ability to dynamically activate HERVs, such as HERV-W (Uleri et al., 2014) and HERV-K (Bhardwaj et al., 2014; O’Carroll et al., 2020). In particular, HIV induces HERV-K transcription and can trigger adaptive immune response against the HERV-K capsid protein (de Mulder et al., 2017). A distinct temporal pattern between HIV and HERV-K activation has been observed in the brains of HIV-infected individuals, demonstrating increased HERV-K activation ahead of spikes in HIV replication in the peripheral blood (Contreras-Galindo et al., 2007) and ahead of clinical symptoms of neurocognitive impairment (Douville and Nath, 2017). HIV-associated motor neuron disease affecting upper and lower motor neurons is likewise escorted by increased HERV-K expression at the onset of neurological symptoms (Bowen et al., 2016). HIV can directly facilitate HERV-K expression, transcript transportation to cytoplasm, and viral particle production (O’Carroll et al., 2020) and regulate anti-viral gene expression through activating (H)ERV promoters (Srinivasachar Badarinarayan et al., 2020). Increased HERV-K env expression in cortical neurons of HIV-infected individuals has been linked with restricting HIV replication in these cells (Bhat et al., 2014). In the long term, however, neuronal HERV-K expression leads to neurite retraction and neuronal death (Dembny et al., 2020), in line with HAND. The antiretroviral therapy against HIV is effective also against HERV-K (Bowen et al., 2016). Overall, the neuropathology induced by HIV and HERV-K might have a certain overlap and is difficult to separate. It might be beneficial to monitor the level of HERV-K within the course of HAND.

Of note, incidence of HIV-associated dementia has reduced threefold after the combination antiretroviral therapy became available (Lawrence and Major, 2002). New medical concerns involve premature aging-related neurocognitive disorders (Robertson et al., 2007). HIV-associated dementia bares certain similarities with that of Alzheimer disease (AD) (Clifford et al., 2009). The neurons of HIV-associated dementia patients contain diffuse Aβ plaques, similar to the early stages of AD (Ortega and Ances, 2014), which could indicate a slower progression of HIV-associated dementia (Fulop et al., 2019).

### Alzheimer’s Disease

Alzheimer’s disease (AD) is a progressive neurodegenerative disorder characterized by gradual cognitive decline. AD can start even decades before the appearance of clinical symptoms (Taylor et al., 2016; Fulop et al., 2018). AD is associated with systemic immune activation and chronic peripheral inflammation (Culibrk and Hahn, 2020). Neuropathologically, AD is characterized by presence of Aβ plaques, Tau neurofibrillary tangles, progressive neuronal death, neuroinflammation, and gliosis in the brain.

Growing evidence points to the role of pathogens, such as HHV-1, HHV-6A, HHV-7 (Lovheim et al., 2015; Readhead et al., 2018), EBV, and CMV (Carbone et al., 2014) in developing sporadic AD. As the worldwide seroprevalence of HHV-1 is around 90% (Wald and Corey, 2007), facilitating factors essentially contribute to the probability of HHV-1 triggering AD (Looker et al., 2015). HHV-1, HHV-6A, and HHV-6B viral glycoproteins can bind β-amyloid oligomers and accelerate Aβ plaque deposition (Eimer et al., 2018).

HERV-H, HERV-K, HERV-L, and HERV-W are transcriptionally active in the brains of AD patients (Johnston et al., 2001; Sun et al., 2018; Dembny et al., 2020). This activation could be directly mediated by HHV-1, HHV-3, and HHV-6 (Ruprecht et al., 2006; Brudek et al., 2007; Tai et al., 2009), or by heterochromatin relaxation and loss of epigenetic host control over HERVs (Sun et al., 2018), increasing DNA damage and expression of pluripotency-related genes (Frost et al., 2014). Upregulation of ERV-K family member in a streptozotocin murine model of sporadic AD was linked with upregulation of immune response genes and downregulation of genes involved in histone modifications and transmembrane transport and associated with cognitive impairments in contextual fear memory and spatial learning (Sankowski et al., 2019). HERV-K (HML-2) transcripts containing a motif 5′-GUUGUGU-3′ contribute to neuronal death and microglial accumulation associated with AD via TLR8 activation (Dembny et al., 2020). ERVs can be transmitted between neurons in the brains of AD patients, packed into an ARC viral-like capsid protein, which is overexpressed in AD patients and is associated with Aβ production (Wu et al., 2011).

### Multiple Sclerosis

Multiple sclerosis (MS) is a neurodegenerative and neuroinflammatory CNS disease characterized by multifocal demyelinating lesions in the brain and spinal cord leading to progressive physical and cognitive disabilities. Development of MS has been associated with viral infections and activation of HERVs (Alvarez-Lafuente et al., 2008; Kriesel et al., 2017; Morandi et al., 2017; Gruchot et al., 2020).

Among viruses, higher transcription levels of HHV-3 and HHV-6 have been found in the CSF of individuals suffering from MS (Mancuso et al., 2007; Alvarez-Lafuente et al., 2008). Also, coronaviruses have been detected in the CNS of MS patients (Burks et al., 1980). EBV has been even suggested as a trigger for MS that activates HERV-W, which then sustains the disease (Mameli et al., 2012).

HERV-W is also the main HERV associated with MS pathology. Further, the expression level of HERV-W in the brain of MS patients correlates positively with the severity of disability and disease progression (Sotgiu et al., 2010). HERV-W and its env transcript and protein are upregulated in the brains (Perron et al., 1997; Antony et al., 2004) as well as in the peripheral blood and serum of MS patients (Garson et al., 1998; Perron et al., 2012a). HERV-W can be activated in MS by EBV (Mameli et al., 2012) and HHV-1 (Ruprecht et al., 2006; Marrodan et al., 2019). HHV-1-triggered HERV-W transcription occurs in immune cells central to the MS pathology, such as B cells, microglia, astrocytes, and macrophages (Ruprecht et al., 2006; Marrodan et al., 2019). HERV-W env protein activates dendritic cells and boosts T helper lymphocyte type-1 (Th1) immune response, acting as a PAMP. It stimulates pro-inflammatory anti-viral response by binding TLR4 and CD14 (Rolland et al., 2006; Saresella et al., 2009). Upon binding the TLR4 on oligodendroglial precursor cells, HERV-W env stimulates release of pro-inflammatory cytokines, inducible nitric oxide synthase, and formation of nitrotyrosine groups leading to reduction of myelin expression in MS lesions (Kremer et al., 2013). In addition to the above, HERV-W env is a powerful superantigen linked with demyelination in MS (Perron et al., 2001; Rolland et al., 2005), perhaps associated with molecular mimicry with myelin oligodendrocyte glycoprotein (MOG) (do Olival et al., 2013; de Luca et al., 2019). Accordingly, treatment with HERV-W env antibody can effectively rescue myelin expression (Kremer et al., 2015).

Mechanistically, HERV-W env protein has been shown to induce microglial polarization and closely associate with myelinated axons in MS lesions ultimately leading to structural damage of these axons (Kremer et al., 2019). HERV-W env, encoded from a full-length provirus at locus 7q21.2, gives rise to a syncytin glycoprotein (Blond et al., 2000; Mi et al., 2000), the expression of which, similar to HERV-W env, is increased by threefold in the brain tissue of MS patients compared with the controls (Antony et al., 2004; van Horssen et al., 2016). HERV-W env and syncytin expression is confined to immunologically active cells, including cells resembling activated glia and phagocytic macrophages at acute and chronic MS demyelinating lesions (Antony et al., 2004; van Horssen et al., 2016). Syncytin activation leads to a myriad of MS-associated pathology, such as pro-inflammatory profile in astrocytes, interleukin-1β (IL-1β) production, cellular protein oxidation, inhibition of oligodendrocyte maturation, myelin damage, and antagonization of remyelination up to neurobehavioral deficits (Antony et al., 2004).

The central role of HERV-W env in MS neurodegeneration has led to the development of a specific monoclonal antibody, Temelimab (GNbAC1) (Curtin et al., 2012), an agent currently being tested in clinical phase II (ClinicalTrials.gov identifier: NCT02782858).

### Amyotrophic Lateral Sclerosis

Amyotrophic lateral sclerosis (ALS) is a neurodegenerative disease, characterized by progressive loss of cortical and spinal motor neurons. While majority of ALS cases are sporadic, mutations in certain genes, such as trans-activation responsive (TAR) DNA-binding protein 43 (TDP-43), have been associated with ALS development (Yousefian-Jazi et al., 2020).

That ALS might be linked with viral infections, comes from finding HIV and HTLV-1 presence in the brains of ALS patients (Verma and Berger, 2006). Further, antiretroviral therapy of HIV-infected individuals with ALS-like syndrome reverses the symptoms related to ALS (Moulignier et al., 2001). The CSF of ALS patients negative of HIV, contains viral reverse transcriptase at levels seen in HIV-infected individuals (MacGowan et al., 2007; McCormick et al., 2008). This has led to investigation of HERVs and revealed the central role of HERV-K among the HERVs in ALS pathogenesis. HERV-K pol, gag, and env are all transcriptionally active in the prefrontal, sensory, motor, and occipital cortex of ALS patients (Douville et al., 2011; Li et al., 2015) and HERV-K env additionally in spinal neurons of sporadic ALS patients. Higher serum IgG and IgM reactivity toward HERV-K gag is also characteristic to ALS patients (Li et al., 2015). HERV-K in ALS can be activated by several mechanisms and occur from distinct cytogenic loci (at 7q36.1) (Douville et al., 2011). These mechanisms include neuronal injury and neuroinflammation through interferon-stimulated response elements in the viral promoter (Gonzalez-Hernandez et al., 2014; Manghera et al., 2016). Once activated, neuronal HERV-K upregulation contributes to sustained neuroinflammation through promoting nuclear translocation of IFN regulatory factor 1 (IRF1) and NF-κB isoforms p50 and p65 (Manghera et al., 2016). Also, TDP-43 activates HERV-K upon binding its DNA (Li et al., 2015). HERV-K and TDP-43 expression in ALS are strongly correlated (Douville et al., 2011). HERV-K env expression leads to neurite retraction, beading, and neurodegeneration (Chen et al., 2014; Li et al., 2015).

### Schizophrenia Spectrum Disorders

Schizophrenia is a neuropsychiatric and neurodevelopmental disorder characterized by episodes of psychosis, hallucinations, and delusions. Disease typically starts in young adulthood and is strongly affected by genetic background and environmental factors (Owen et al., 2016). The neurobiology behind schizophrenia is poorly understood.

The likelihood of developing schizophrenia is increased by infections (most significantly evidenced with studies on HHV-2) (Arias et al., 2012), subclinical inflammation (Frydecka et al., 2018), and variation within brain-associated and immune genes (Schizophrenia Working Group of the Psychiatric Genomics, 2014). Altered immune response and lasting inflammatory dysregulation of the nervous system are associated with chronic stress exposure (Pearce et al., 2019; Nettis et al., 2020). Upregulated immune response genes induce hyperactivation of LINE-1, which is common in schizophrenia patients (Bundo et al., 2014). Increased retrotransposition of LINE-1 is found in the neurons of prefrontal cortex, affecting intragenic regions and synaptic genes (Bundo et al., 2014).

In addition to LINE-1, expression of several HERV families, such as HERV-K, HERV-W, and HERV-H, has been shown to be dysregulated in the brains, cerebrospinal fluid, and blood of schizophrenia patients (Perron et al., 2012b; Li et al., 2019; Mak et al., 2019). This could involve activation of distinct HERV loci. For example, activated HERV-W env transcripts in schizophrenia have been shown to differ from these activated in bipolar disorder or MS. Combined with HERV-W copy number differences between schizophrenia patients and healthy controls, this might point to perinatal HERV-W activation (for instance by infections such as influenza), potentially leading to inflammation and subsequent neurotoxicity (Limosin et al., 2003; Perron et al., 2008, 2012b). HERV-W env protein expression in developing hippocampus was recently shown to alter the N-methyl-d-aspartate receptor (NMDAR)-mediated synaptic organization and plasticity. This was associated with defective glutamate synapse maturation, behavioral impairments, and psychosis (Johansson et al., 2020). Apart from HERV-W, lower DNA methylation levels at HERV-K sequences in peripheral blood have been shown to be specific to early stages of schizophrenia (Mak et al., 2019).

### Neuropathogenesis of SARS-CoV-2

Highly pathogenic severe acute respiratory syndrome coronavirus-2 (SARS-CoV-2) is a single-stranded RNA virus from the coronavirus’s family (Su et al., 2016).

SARS-CoV-2 enters host via its immunogenic spike glycoprotein binding to ACE2 receptor on endothelial and smooth muscle cells (Lan et al., 2020; Kaneko et al., 2021). Resultant coronavirus disease 2019 (COVID-19) mainly presents as an acute respiratory disease; however, also neurological symptoms have been reported, including dizziness, headache, encephalitis, seizures, intracerebral hemorrhage, and stroke (Benger et al., 2020; Guadarrama-Ortiz et al., 2020; Huang et al., 2020; Lu et al., 2020; Moriguchi et al., 2020). In addition, neuromuscular and autoimmune complications are associated with COVID-19. These include most frequently Guillain Barré syndrome but also Miller Fisher syndrome, polyneuritis cranialis, acute myelitis, oculomotor paralysis, and Bell’s palsy have been reported (Guadarrama-Ortiz et al., 2020; Katyal et al., 2020). SARS-CoV-2 uses the olfactory nerves and possibly the vagus nerve to access the brain (Guadarrama-Ortiz et al., 2020; Liu et al., 2020) where ACE2 receptor is expressed on neurons and glial cells (Zou et al., 2020). The neuromuscular invasion of SARS-CoV-2 likely involves retrograde axonal transfer of the virus in trans-synaptic pathway and cytokine storm (Katyal et al., 2020).

SARS-CoV-2 viral proteins interact with human proteins that regulate cellular longevity and aging, and are involved in stress response, vesicle trafficking, lipid metabolism, production of reactive oxygen species, RNA regulation, ubiquitin ligases, and mitochondrial activity (Gordon et al., 2020). Impaired lysosomal function combined with inhibition of ubiquitin–proteasome system can cause protein misfolding and protein aggregation in affected cells, including neurons, a common mechanism of many neurodegenerative diseases (Lippi et al., 2020).

Up to one third of COVID-19 patients develop neurological symptoms beyond the acute stage of the disease, mainly manifesting with chronic fatigue syndrome and myalgic encephalomyelitis (Nath and Smith, 2021). That several coronaviruses (CoV-OC-43, CoV-229E, and HCoV) are found in the brains of MS patients or have been associated with MS pathology (Burks et al., 1980; Murray et al., 1992) could indicate MS-like demyelinating neuropathology as a possible long-term complication of COVID-19. Further, it remains to be investigated how SARS-CoV-2 affects the expression of HERVs, LINE-1, and Alu elements and interacts with other viruses and environmental factors.

## Aging

Aging is a progressive deterioration of physiological functions at the cellular, organ, and organism levels eventually leading to senescence. Aging disrupts the balance between the nervous and the immune system and increases risk for various neurodegenerative and neuroinflammatory diseases (Streit et al., 2004; Godbout et al., 2005; Valdes-Ferre et al., 2020).

Aging increases genomic instability (Lombard et al., 2005) by gradual loss of global DNA methylation and region-specific DNA hypermethylation (Jung and Pfeifer, 2015). Increased age-related activation of certain retrotransposon families is found in mice (ERVs) (Odaka, 1975) and Drosophila (LINE-like R2, LTR element gypsy transcripts, and env glycoprotein) (Li et al., 2013). In humans, aging causes profound de-repression of HERV-K, Alu, and LINE-1 elements (Bollati et al., 2009; Cardelli, 2018) with increasing chromatin openness at Alu, SVA, and LINE-1 elements in senescent cells (De Cecco et al., 2013). This affects most significantly evolutionarily younger elements (De Cecco et al., 2013). Transcription levels of HERV-H, HERV-K, and HERV-W change in distinct patterns during human life. HERV-H is highly transcribed in childhood, while HERV-K and also HERV-W transcription increases on reaching higher age (Balestrieri et al., 2015; Autio et al., 2020). ERV activation in aging Drosophila causes shorter lifespan, neurodegeneration, and memory deficits (Li et al., 2013). Similar effects of ERV activation on hippocampal memory and cognitive impairment are observed in mice (Sankowski et al., 2019). Particularly, in combination with chronic inflammation, the effect of HERV activation in aging brain can be detrimental and contribute to neuronal decline (Johnston et al., 2001; Sankowski et al., 2019). In addition to HERVs, LINE-1 hypomethylation has been described in the peripheral blood of elderly individuals (Mahmood et al., 2020). Some of the age-related epigenetic changes, such as those related to Alu methylation, seem to be regulated by longevity-associated genetic factors, including genes involved in nucleotide biosynthesis, metabolism, and signal transduction (Gentilini et al., 2013).

Aging can determine the outcome of interplay between endogenous and exogenous viruses. Interaction of the endogenous murine leukemia virus with the generally non-pathogenic murine togavirus lactate dehydrogenase-elevating virus leads to a fatal and progressive neurological disease in up to 100% of aged mice. This suggests convergence of age-related, genetic, immunological, and viral factors in the development of a neurological disease resembling ALS in humans (Contag and Plagemann, 1989).

## Concluding Remarks

This review brings together studies that have described a role for exogenous viruses and (H)ERVs in CNS pathologies and thereby highlights the interplay between the inherent and the foreign. A contribution of exogenous and endogenous viruses, separately and together, is increasingly evident in common forms of dementia in young (HAND) and elderly population (AD), MS, ALS, and also schizophrenia. In other neurological complications of viral origin, such as SARS-CoV-2, it remains to be seen if and how HERV, LINE-1, and Alu expression may be involved. Viral CNS infections can be early triggers of neuroinflammation; however, if viruses are successfully combated or entered in a latent state, a central role might be attributed to endogenous retroviruses. ERV activation during infections seems to be a common (physiological) mechanism that the host may not be able to control at some point. ERVs can become continuously activated and sustain the inflammatory imbalance. The crosstalk with IFN seems to play an important role here. Facilitating factors that are associated with continuous ERV activation such as aging, stress, and other comorbidities as well as re-awakening of a latent virus, cumulative or opportunistic infections, as seen in immune-deprived conditions, contribute to the progressive neurodegeneration or delayed CNS pathologies. We are only beginning to understand how exogenous viruses in connection with HERVs and other retroelements affect normal aging and development of neurodegenerative diseases and other neuropathologies. The central role of HERV-W in MS pathology has led to its targeting in clinical trials. It remains to be seen, whether other HERVs could provide key targets in other neurodegenerative diseases, such as HERV-K in ALS, to which there is currently no cure.

## Author Contributions

CR designed the manuscript idea, performed literature search, and wrote the manuscript.

## Conflict of Interest

The author declares that the research was conducted in the absence of any commercial or financial relationships that could be construed as a potential conflict of interest.
